# Measuring deliberate reflection in residents: validation and psychometric properties of a measurement tool

**DOI:** 10.1186/s12909-023-04536-2

**Published:** 2023-08-25

**Authors:** Richard H. Blum, Christine L. Mai, John D. Mitchell, Daniel Saddawi-Konefka, Jeffrey B. Cooper, George Shorten, Audrey DunnGalvin

**Affiliations:** 1 Department of Anaesthesia, Harvard Medical School, MA Boston, USA; 2 Department of Anesthesiology, Critical Care, and Pain Medicine, Boston Children’s Hospital, 300 Longwood Avenue, MA 02115 Boston, USA; 3 The Center For Medical Simulation, Charleston, MA USA; 4 Department of Anesthesia, Critical Care and Pain Medicine, Massachusetts General Hospital and Harvard Medical School, Boston, MA USA; 5 Department of Anesthesiology, Pain Management and Perioperative Medicine, Henry Ford Health Systems, Detroit, MI USA; 6 Anesthesia and Intensive Care Medicine, School of Medicine, University College Cork, Cork, Ireland; 7 Insight II SFI Research Centre, Cork, Ireland; 8 Department of Anesthesia and Intensive Care, Cork University Hospital, Cork, Ireland; 9 Early Years and Childhood Studies in the School of Applied Psychology, Cork University Hospital, University College Cork, Cork, Ireland; 10 Department of Paediatrics and Child Infectious Diseases, I.M. Sechenov First Moscow State Medical University, Moscow, Russia

**Keywords:** Reflection, Reflective Practice, Anesthesia Trainees, Assessment, REFLECT Rubric & Validation

## Abstract

**Purpose:**

Reflective capacity is “the ability to understand critical analysis of knowledge and experience to achieve deeper meaning.” In medicine, there is little provision for post-graduate medical education to teach deliberate reflection. The feasibility, scoring characteristics, reliability, validation, and adaptability of a modified previously validated instrument was examined for its usefulness assessing reflective capacity in residents as a step toward developing interventions for improvement.

**Methods:**

Third-year residents and fellows from four anesthesia training programs were administered a slightly modified version of the Reflection Evaluation for Learners’ Enhanced Competencies Tool (REFLECT) in a prospective, observational study at the end of the 2019 academic year. Six written vignettes of imperfect anesthesia situations were created. Subjects recorded their perspectives on two randomly assigned vignettes. Responses were scored using a 5-element rubric; average scores were analyzed for psychometric properties. An independent self-report assessment method, the Cognitive Behavior Survey: Residency Level (rCBS) was used to examine construct validity. Internal consistency (ICR, Cronbach’s alpha) and interrater reliability (weighted kappa) were examined. Pearson correlations were used between the two measures of reflective capacity.

**Results:**

46/136 invited subjects completed 2/6 randomly assigned vignettes. Interrater agreement was high (k = 0.85). The overall average REFLECT score was 1.8 (1–4 scale) with good distribution across the range of scores. ICR for both the REFLECT score (mean 1.8, sd 0.5; α = 0.92) and the reflection scale of the rCBS (mean 4.5, sd 1.1; α = 0.94) were excellent. There was a significant correlation between REFLECT score and the rCBS reflection scale (*r* = .44, *p* < 0.01).

**Conclusions:**

This study demonstrates feasibility, reliability, and sufficiently robust psychometric properties of a modified REFLECT rubric to assess graduate medical trainees’ reflective capacity and established construct/convergent validity to an independent measure. The instrument has the potential to assess the effectiveness of interventions intended to improve reflective capacity.

**Supplementary Information:**

The online version contains supplementary material available at 10.1186/s12909-023-04536-2.

## Introduction

Reflective capacity, one aspect of reflection, has been defined as “critical analysis of knowledge and experience to achieve deeper meaning and understanding” [1]. It is considered an important, perhaps critical, component of successful learning in medicine. After a challenging event, the clinician internalizes the experience by thinking about what happened, how it felt, how they behaved, and the outcomes. This is a deliberate way of thinking about experiences; to learn from mistakes, to identify skills and strengths, and to develop options and actions for change. This is essential to promote a lifelong process of learning and development [2]. Key terms and definitions of reflection are provided in Supplementary File Section 1. In an earlier study, we observed that anesthesia residents did not progress in the quality of their reflection following a simulation assessment experience from the first to third years [3]. This is not surprising given that, in graduate medical education, little has been done to teach how to reflect deliberately.

We sought to learn more about the reflective capacity of residents in an absolute sense and about characteristics that might correlate with or enhance deliberate, productive reflection. If levels of reflection are insufficient to promote optimal learning, interventions are needed to elevate reflection in practice. To assess interventions, a reliable, valid measurement tool is required. We report here on a study that builds on our earlier pilot study, which applied an established rubric used for medical students with the aim of determining if a similar assessment tool would be reliable for residents. We used a cohort of anesthesia residents as a sample population.

Our specific aims were 1) to establish the feasibility, reliability, psychometric properties, and applicability to a different trainee population (residents) of the REFLECT Score for the assessment of reflective capacity (RC) [4] and 2) to assess the construct/convergent validity [5] of measured RC via REFLECT by comparing scores to those on the RC subscale of the Cognitive Behavioral Survey: Resident Level (rCBS) [6].

## Methods

### Study design

We carried out a prospective observational study designed to establish the feasibility, scoring characteristics, reliability, validation, and generalizability of a modified REFLECT scoring rubric for the assessment of anesthesia trainees’ reflective capacity. A second assessment method (rCBS) was used to examine construct validity.

### Subjects

After receiving an exemption from the institutional review board in 2019 (see declaration section), 136 third-year anesthesia residents (CA-3; PGY4) and fellows (PGY5) from four Accreditation Council for Graduate Medical Education (ACGME) accredited anesthesia training programs affiliated with Harvard Medical School [Boston Children’s Hospital (BCH), Beth Israel Deaconess Medical Center (BIDMC), Brigham and Women’s Hospital (BWH) and Massachusetts General Hospital (MGH)] were invited to participate in the study via email solicitation at the end of the 2019 academic year.

### Measures

#### Evaluation of reflective capacity

Reflective capacity was assessed and scored using the Reflection Evaluation for Learners’ Enhanced Competencies Tool (REFLECT), which was developed and validated by Wald and colleagues [4]. The slight modifications related to the wording or description of levels 1 and 2 of two of the criteria. For example, for Analysis instead of ‘little or no analysis’ for Levels 1 and 2, we used the terms ‘little or no analysis’ = 1 and ‘some analysis’ = 2. This helped the raters to distinguish between these two levels.

Those modifications were used in our earlier pilot study [7] and produced excellent internal reliability (Supplementary File Section 2).

Six “vignettes” (descriptions of a simulated scenario of an anesthetic case) were developed and included some element of suboptimal care using predefined design elements (Supplementary File Section 3 for sample vignette with associated design elements; details of how vignettes were constructed are given in reference #[7]). Each trainee was randomly assigned two vignettes in a random order and instructed to answer three questions as if they were the primary anesthesia provider described in each of the vignettes: 1. What happened? 2. What are your thoughts and feelings about why this event happened? 3. What could you have done differently? Each response was scored on: 1. Presence (evidence of writer is prevalent in the text with use of the personal pronoun), 2. Description of conflict or disorienting dilemma (the disorienting dilemma/event is clearly formulated), 3. Attending to emotions (emotions are noted and some insight is gained), 4. Analysis and meaning making (the event is analyzed through more than one perspective and meaning is drawn), and 5. Writing spectrum (overall style is detailed and compelling with the writer adding their own insights, experiences, thoughts or impacts). Each trainee was randomly assigned to complete two of the six study vignettes that were created by the study team. The responses were scored by two trained raters using the Modified REFLECT Rubric, which sets out the dimensions of the construct to be scored and benchmarks for each that represent advancing levels of reflection: Level 1—Habitual action (Nonreflective), Level 2—Thoughtful action or introspection, Level 3 – Reflection, and Level 4—Critical reflection. Each of these levels, and the five criteria that constitute each, were defined by Wald and colleagues [4] with minor modifications.

We asked respondents to answer two additional questions: 1. Realism: “To what extent do you think your written responses about your thoughts and feelings (not specific clinical actions) are similar to how you’d respond if this were your actual clinical case?” with a response scale from 1 = not at all similar to 7 = extremely similar, and 2. Importance: “Reflection is important to my professional development,” with a response scale from 1 = not at all important to 5 = very important.

#### Evaluation of cognitive, metacognitive, and experiential learning

The Cognitive Behavior Survey: Residency Level (rCBS) measures cognitive, metacognitive, and experiential learning [6]. The measure consists of 120 statements (items) that describe specific learning behaviors or attitudes. Residents completed the entire survey, rating the degree of their agreement with each statement by means of a seven-point rating scale, from 1 = “strongly disagree” to 7 = “strongly agree.” The items fall into one of seven scales: memorization, conceptualization, reflection, independent learning, critical thinking, meaningful learning experience, and attitude toward educational experience. Each of the seven rCBS scales represents a separate construct that contributes to learning behavior [8]. The measure has been shown to have good scale reliability and construct validity in a sample of medical residents [6]. The primary focus of the rCBS for this study was the Reflection Scale (comprising 12 items), where respondents were asked about their level of agreement with statements including: I find time to reflect on how past clinical events relate to one another; and I find time to think about the relevance of new material by hypothesizing how it might guide my behavior in future clinical situations.

### Rater training

Two raters (anesthesiology trainees, year one and six of the Irish National Training Program) underwent training during three, 90-min face-to-face sessions with an expert in psychometric evaluation (ADG) (Supplementary File Section 4). Since we deliberately developed vignettes of proto-typical events/dilemmas that residents would encounter in anaesthesia, the knowledge level of the trainees was appropriate. Furthermore, a subset of the rating (30%) was checked by ADG for quality and consistency. The training included a detailed description of the five categories of the Modified REFLECT Rubric and instruction on how to score each category and included review of vignettes until the reviewers agreed with the instructor scoring with < 0.5 on a 10-point scale. For this study, we created a manual and a set of “Rules of Thumb” to aid in scoring (Supplementary File Section 3).

### Data preparation

We used the mean of the five criterion scores of the Modified REFLECT Rubric as the primary reflection measure, termed the REFLECT Score. Scores for each vignette were analyzed using descriptive statistics (mean and standard deviation). Because the Pearson correlations between vignettes were high (vignettes 1–6 ranged from *r* = 0.83 to 0.91) we used the mean of the two vignettes assigned to each subject for analysis and refer to it as that subject’s REFLECT Score. The data on REFLECT Score and rCBS Reflection Scale Score (split by gender) was tested for normality of distribution using Kolmogorov-Smirnova (*p* > 0.1 with a Lilliefors Significance Correction), Shapiro–Wilk (*p* > 0.1), and an inspection of the Q_Q normality plots, with assumptions for normality met. For the REFLECT Scoring, only cases that completed two scenarios were included in the analysis. rCBS data were only included if fewer than < 20% of the items were missing.

### Scale reliability

Scale reliability for the REFLECT Score and the rCBS Reflection Scale Score were examined using Cronbach’s alpha. The accepted value of Cronbach's alpha is 0.7; however, values above 0.6 are also acceptable [9].

### Profile of the participants and scenarios

Demographic and clinical data and any differences in scores on REFLECT Score and on rCBS Reflection Scale Score according to participant characteristics (gender, training level and program) were examined with univariate analysis.

### Interrater reliability

A weighted kappa was used to assess agreement between raters. Values greater than 0.75 were considered excellent agreement, below 0.40 were considered to represent poor agreement and values between 0.40 and 0.75 were considered to represent fair to good agreement beyond chance [10, 11].

### Construct/convergent validity

Since each of the seven rCBS scales represents a construct about learning behavior, with some more likely to be correlated than others, we followed Streiner and Norman and Mitchell’s approaches to investigate construct/convergent validity.

We hypothesized that the REFLECT Score would have:

A) a significant positive correlation with the rCBS Reflection Scale;
B) a significant positive correlation with trainee’s perception of how similar their written responses were compared with what they would have been if it were their actual case (for brevity in the manuscript, we named this variable ‘Realism’);
C) and a significant positive correlation with the survey question “Reflection is important to my professional development” (for brevity in the manuscript, we named this variable ‘Importance’).

To further examine the presence of construct validity for the REFLECT Scoring, we created a variable designating high, moderate and low reflection scorers according to the reflection subscale on the rCBS. High reflection scorers were defined as those whose reflection scores were among the top 25^th^ percentile, low reflection scorers were those whose scores were among the bottom 25^th^ percentile and moderate were those between these two points. We used a univariate analysis of variance (ANOVA) to determine if a significant difference could be found for the REFLECT Score (dependent variable), according to the three groups (low, moderate, high) for the reflection scale or the rCBS. This analysis also helps to better understand differences in high and low performers with practical implications for support and training.

### Data collection and management

Study data were collected and managed using REDCap electronic data capture tools hosted at Boston Children’s Hospital. Subjects were sent a link to a REDCap survey that included demographic data, clinical vignettes and the rCBS survey. Results of the statistical tests were considered significant when the probability of making a Type I error was less than 0.008% adjusting for multiple testing using the conservative Bonferroni method.

All analyses were performed in PASW for Windows version 27 (SPSS Inc., Chicago, IL).

## Results

### Participant profile

Of the 136 trainees invited to participate, 47 responded: one respondent completed only one of the vignettes and was removed from subsequent analyses. The final (analyzed) sample consisted of 46 trainees who completed two of the six possible vignettes for analysis for their REFLECT Scores alone. Six of the respondents did not complete the rCBS either fully or in part (> 20% of items missing), therefore data comprised 41 completed responses for analysis of the rCBS alone, or for the rCBS paired with another measure e.g., REFLECT Score. Of those who provided their gender (*N* = 39) and training level (*N* = 42), 51% were male, and all were either third-year anesthesia residents (CA-3) or fellows (Table 1). Of the four programs that participated, the majority of participants were recruited from BWH and BIDMC (70%), with the remainder from MGH and BCH*.* There were no significant differences (all *p* > 0.1) for gender, training, or program on the rCBS Reflection Scale (t = 0.705; *F* = 1.11; *F* = 1.15, respectively), or the REFLECT Score (t = 1.41; *F* = 1.39; *F* = 0.453, respectively). Therefore, below we present the pooled data for the sample.

**Table 1:** Demographics: Trainee program and training level

Program ^a^		Training Level ^a^	
	Total N (%)	CA-3 *N* = 18 (45%)	Fellows *N* = 26 (55%)
BCH	5 (11)	0	5
BIDMC	16 (36)	8	8
BWH	15 (34)	7	8
MGH	8 (18)	3	5

*BCH* Boston Children’s Hospital, *BIDMC* Beth Israel Deaconess Medical Center, *BWH* Brigham and Womens’ Hospital, *MGH* Massachusetts General Hospital

^a^*N* = 44 (96%) of total sample (*N* = 46) answered this question

### Interrater reliability

The two raters were blinded to the identity of the trainees whose responses they scored; level of expertise and individual vignette responses were randomly allocated (i.e. raters did not score different vignettes’ responses of the same individual trainee in sequence, to avoid priming bias). A trainee’s responses for a given vignette were presented to raters and evaluated as a unit, according to the REFLECT Score. The weighted kappa for agreement between raters, averaged across the scenarios, was κ = 0.85. The average of two raters’ scores were used in the subsequent analyses. The average time taken to rate one vignette (including reading time) was approximately 10 min.

### Reliability of the measures

Internal consistency reliability for both the REFLECT Score (five items) and the Reflection Scale of the rCBS (12 items) were excellent (α = 0.92 and 0.94 respectively).

### Participant scores on measures

The mean REFLECT Score (scale of 1–4) was 1.8 (SD 0.5). Participants obtained the greatest mean scores for the Dilemma (2.0) and Analysis (2.1) items of the Modified REFLECT Rubric (Table 2). The lowest mean scores were for the Emotions (1.3) and Presence (1.6) scales. The greatest mean scores on the rCBS (scale of 1–7) were for the Conceptualization and Attitude subscales (both 5.1), with the lowest scores obtained on the Memorization and Independent Learning (both 4.3). The mean score for the Reflection subscale was 4.5 (SD 1.1); our subsequent analysis focuses on this domain.

**Table 2:** Means (SD) for REFLECT Scores and rCBS Reflection Scale Scores

**REFLECT Score**^a^							**rCBS**^**b**^
Item	Presence	Dilemma	Emotions	Analysis	Writing	Total Score	Reflection Scale
Mean (SD)	1.6 (0.6)	2.0 (0.6)	1.3 (0.4)	2.1 (0.6)	1.8 (0.5)	1.8 (0.5)	4.5 (1.1)

^a^Response Scale 1 to 4 (4 is best)

^b^Response Scale 1 to 7 (7 is best)

For the additional questions, the mean score for “Realism” was 5.2 (SD 1.0) on a 7-point response scale and the mean score for “Importance” was 4.2 (0.9) on a 5-point response scale (Table 3).

**Table 3:** Mean REFLECT, rCBS Reflection Scale, “Realism” and “Importance to Practice” scores by vignette and associated Pearson correlations

	*n*		Total	Vignette Number					
				1	2	3	4	5	6
REFLECT Score	41	*Mean (SD)*	1.8 (0.5)	1.9 (0.6)	1.7 (0.5)	1.8 (0.5)	2.0 (0.4)	1.7 (0.6)	1.7 (0.4)
Pearson r Correlations									
rCBS Reflection Scale Score	41	4.5 (1.1)	.44^b^	.67^a^	.30^a^	.21 (NS)	.49^a^	.58^b^	.28^a^
“Realism”	41	5.2 (1.0)	.62^b^	.58^a^	.42^a^	.48^a^	.58^a^	.65^b^	.43^a^
“Importance”	41	4.2 (0.9)	.28^a^	.52^a^	.20 (NS)	.23 (NS)	.55^b^	.59^b^	.17 (NS)

^a^Correlation is significant at the 0.05 level (1−tailed)

^b^Correlation is significant at the 0.01 level (1−tailed)

### Construct validity

As hypothesized, we found a significant positive correlation (Table 3) between the REFLECT Score and the rCBS Reflection Scale (*r* = 0.44, *p* < 0.01). There were significant positive correlations for “Realism” (Mean 5.2, SD 1) and “Importance” (Mean = 4.0, SD 0.8) and the REFLECT Score (*r* = 0.62, *p* < 0.01) and (*r* = 0.28, *p* < 0.01) respectively (Table 3).

At the level of individual vignettes, there was statistical significance for all vignettes except vignette 3, which had a positive trend but did not reach statistical significance. The strongest correlations were for vignettes 1, 4, and 5 (Table 3).

**Fig. 1:**
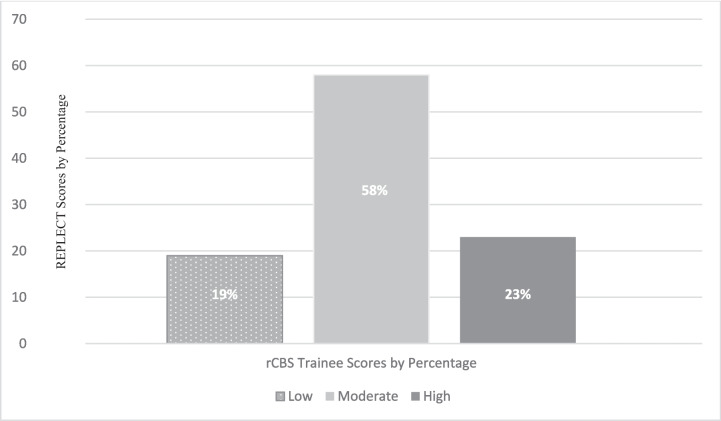
Percentage Distribution for the 3 Groups of REFLECT Scores (low, moderate, high)

To examine the ability of the REFLECT scoring to distinguish lesser from greater scoring trainees, we used a univariate ANOVA to determine if there was a significant difference on the REFLECT Score (dependent variable), according to the three groups on the rCBS Reflection Scale (low, moderate, high scorers). Figure 1 shows the percentage distribution and mean scores across the three groups.

**Fig. 2:**
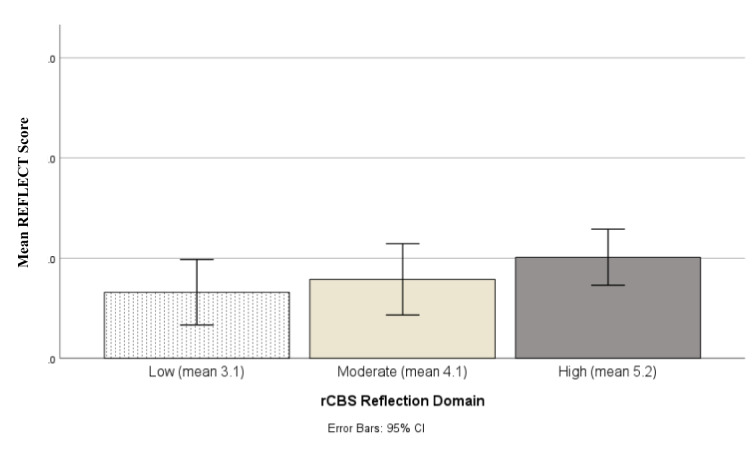
Mean REFLECT Scores (dependent variable) vs. to the rCBS Reflection Scale scores (low, moderate, and high)

The ANOVA indicated a significant difference overall in mean scores on the REFLECT Score [*F* = 5.52, *p* < 0.01] that mapped onto the three rCBS scoring groups (Fig. 2). Post hoc comparisons using the Tukey test showed that the significant differences lay between Low Scorers and High Scorers (mean difference -0.73, *p* < 0.01) and between Moderate Scorers and High Scorers (mean difference -0.53, *p* < 0.05).

Post hoc comparisons using the Tukey test showed that the significant differences lay between Low Scorers and High Scorers (mean difference -0.73, *p* < 0.01) and between Moderate Scorers and High Scorers (mean difference -0.53, *p* < 0.05).

## Discussion

Using a different cohort and examining validity more robustly, the findings in this study add further evidence to establish the feasibility, reliability, psychometric properties, and generalizability of a modified REFLECT scoring rubric (i.e. the REFLECT Score) for the assessment of graduate medical trainees’ reflective capacity (RC) [4] and to establish the construct/convergent validity [5] of measured RC via REFLECT [6]. Reflective capacity (RC) is regarded by many as an essential characteristic for professional competence; teaching to enhance RC has been integrated into undergraduate, postgraduate, and continuing medical education [12]. However, more evidence is needed to inform curricula beyond the theoretical, to allow for a valid examination of learning effectiveness. A valid instrument for RC assessment enables its standardization and use with other elements in competency-based education [13].

Our earlier pilot study, which was conducted with a cohort of 29 anesthesia residents at the University College Cork (UCC), Ireland, introduced the REFLECT Score, which had been previously validated for medical students only [7]. One study involved medical interns, but none have involved more advanced trainees [14]. We demonstrated that meaningful clinical vignettes could be written, producing a wide scoring range, that raters could be trained to produce strong inter-rater agreement and established a baseline of RC scores for a small group of anesthesia trainees in a single hospital.

Construct validity establishes that a measure ‘behaves’ in a manner which is consistent with theoretical hypotheses and represents how well scores on the instrument are indicative of the theoretical construct; it is central to validity research [15]. Construct validity is typically demonstrated by comparing the test or measure to other tests that measure similar qualities to see how highly correlated the two measures are. In the present study, as hypothesized, we found a significant positive correlation between the REFLECT Score and the rCBS Reflection Scale Score. Our analysis also showed significant differences between Low Scorers (bottom 25^th^ percentile) and High Scorers (top 25^th^ percentile). This adds to the correlational analyses findings, which together suggest that the REFLECT Score a) has construct/convergent validity, and b) may be used to correctly distinguish high and low scorers with high and low reflection abilities in a sample, using short responses to a previously developed vignette. Mitchell (2009) also found significant differences for the rCBS Reflection Scale Scores between high (top 20%) and low scorers (bottom 20%). In addition, varied scoring over almost the full range further supported the REFLECT Score as a useful measure of RC, a central aim of the present study.

The response to the question “*To what extent do you think your written responses about your thoughts and feelings (not specific clinical actions) are similar to how you’d respond if this were your actual clinical case?”* was relatively strong (5.2 on a scale of 1–7). This question may provide a surrogate for ‘realism’ or how deeply the respondent engages and identifies with the story. The practical implications suggest that vignette stories are meaningful for respondents, although we cannot state with any certainty that their response would be the same to a real event that they themselves experienced. We hope to learn more about this issue in our next study, in which respondents will reflect on a personal event of their choosing (in addition to pre-prepared vignettes).

The significant correlation found between participant perception of the importance of RC to clinical practice and the rCBS Reflection scale and the REFLECT Score indicates that a positive perception of the value of RC may affect how much they value the role of RC, or how they perceive the benefits of RC to their practice, which in turn may impact how well they actually reflect. In effect, if a resident is not convinced of the benefit of RC to practice, they may not fully engage in the process.

Our findings lead us to several conclusions that helped us in designing our larger study, the results of which are currently being evaluated. First, the REFLECT scoring rubric is more than sufficiently reliable for our purposes and generates a wide range of scores across a sample, a property that is desirable in a behavioral instrument. We believe the rating reliability is the result of the rigorous rater training received by the two domain experts who coded each vignette.

Secondly, the REFLECT score of each of the six vignettes correlates moderately with the rCBS Reflection Scale Score, providing some evidence of the ability of the vignettes to elicit RC. Some of the vignettes did not demonstrate acceptable convergent validity, typically considered to be a correlation of at least > 0.5 with an instrument measuring the same construct [5]. We are using the three vignettes that provided the strongest convergent validity in our current larger study of RC. In a follow-up analysis, we aim to qualitatively compare the components across vignettes that may account for these differences.

We also demonstrated the feasibility of operating a user-friendly, secure online platform to administer the REFLECT instrument and have it rated efficiently. In our prior study, approximately 10—20 min were required to complete each vignette; probably due to more rater training and experience, in the current study, raters rated a vignette in approximately 10 minutes.

Much has been written about reflection and its assessment. The term itself has no single agreed upon definition, yet it is generally accepted that thinking about one’s specific clinical experiences to learn how to improve performance in any medical specialty is an important, perhaps vital process for all learners. Evidence shows that, if medical students are unclear as to the purpose of reflection and do not see educators modelling reflective behaviors, they are likely to undervalue this important skill regardless of the associated learning and development opportunities embedded in the curriculum [16]. Based on interpretation of writings and studies of reflection in general and on our own prior work, graduate medical education programs would do well to learn more about how much and how well trainees reflect on their clinical experiences. REFLECT scores overall were in the range of “Thoughtful Action or Introspection” (2 on 4-point Likert scale) to “Reflection” (3 on the 4-point scale). If, as our findings suggest to us, reflective capacity in many residents is on the lower end of the scale, taking deliberate action to improve it may be meaningful for those that may benefit.

We note that there is controversy about whether measurement of reflection in any of its forms is desirable or appropriate [17, 18]. Yet, we were led to use REFLECT in this way because of our belief that something cannot be improved if it cannot be measured.

Some interventions to improve reflective performance have been proposed but there is no robust assessment of a practical measurement tool tested extensively in residents. That is required to determine which interventions are effective, if any. The evidence we are creating so far suggests that the REFLECT tool could be such an instrument for that purpose. From the opinions of the residents in this pilot, the instrument itself might also have properties of a useful intervention as well.

In addition to Wald’s studies demonstrating REFLECT’s acceptable psychometric properties, Brown used the original REFLECT scoring in 4^th^ year medical students with good reliability (IRR = 0.8 among five well-trained raters) [19]. Grierson, et al. using REFLECT, did not achieve robust reliability for their raters. As we noted previously, that may be because their training was not as extensive nor did they seem to test the raters for reliability before the actual rating [17].

### Limitations

Because we now have successfully administered the REFLECT instrument in two different countries and across four independent anesthesia training programs, we believe that the reliability and validity data presented in this study provide preliminary support for REFLECT as a useful tool to measure RC. However, our samples were relatively small and further investigation is necessary to confirm these results and to provide evidence of the applicability of REFLECT to other and more varied resident populations to ensure they are generalizable.

There may be selection bias since only 47/136 elected to participate. We can only speculate on which direction that bias might be; it is tempting to think the more reflective agreed to participate, but the overall level of reflection suggests that may not be the case. The small number of vignettes completed per resident (2) is also a limitation and we did not include a personal vignette in this study. This limits our ability to have greater certainty in some of our analyses including differences in scores across vignettes and differences in some of the correlations with REFLECT scores and rCBS survey data. We acknowledge that, because all self-report measures and assessments were evaluated at only one time point, they are vulnerable to confounding factors that may have been present. The division of scores into low, moderate and high groups is relative to the scores of the subjects; it does not imply the scores are absolutely rated based on an optimal level of reflection. Also, the fundamental lack of agreement on the definition of reflection is a limitation of all studies on the construct. However, we clearly provided a definition based on prior evidence-based research. Further studies add information to the discussion of the meaning and importance of reflection in medical education.

While rater training requires only approximately 4.5 h of time commitment, it could be a limitation for practical application of this instrument for reflection assessment. We have already made it more efficient, entirely remote and with limited instructor involvement.

## Conclusions and future work

Schon wrote about the ‘messiness of professional practice’ in which uncertainty, hesitation and misgivings are part of the learning experience [13, 20]. RC can support medical practitioners in dealing with the everyday challenges inherent in practice while also promoting well-being in themselves and their patients [21]. Furthermore, integrating a transdisciplinary RC curriculum may enhance cooperation and a shared perspective across (for example) operating room nurses, surgeons, anesthesiologists and related clinicians, with benefits both for practice and for patient care.

We are currently evaluating findings from a much larger cohort of residents to learn if RC is different between training programs, level of training, gender, and vignette type. Together with providing further evidence of reliability, validity, and generalizability of the REFLECT Score and rater training protocol, our aim is also to understand the key variables influencing RC. This will support the longer-term goal of development and integration of RC within education and practice throughout the professional life cycle. Our approach may be appropriate for technology-enhanced learning and to deliver targeted educational programs using real-life scenarios and high-fidelity simulation sessions. We hope our continuing work will help to inform the impact of RC on patient care amongst practitioners, educators, and researchers.

### Supplementary Information

**Additional file 1:** **Supplementary File Section 1.** Key terms and definitions of reflection. **Supplementary File Section 2.** Modified REFLECT Scoring Rubric. **Supplementary File Section 3.** Vignette with Essential Design Elements, Trainee Response, “Rules of Thumb” for Scoring and Qualitative and Qualitative Scoring. **Supplementary File Section 4.** Steps in Rater Training.

## Data Availability

The anonymized datasets used in this study are available from the corresponding author on request.
