# Supplementary material for: Measuring deliberate reflection in residents: validation and psychometric properties of a measurement tool
Source: BMC Med Educ. 2023 Aug 25;23:606. doi: 10.1186/s12909-023-04536-2 (PMC10463616; doi:10.1186/s12909-023-04536-2)
Supplement: Supplementary file 1 — Additional file 1: Supplementary File Section 1. Key terms and definitions of reflection. Supplementary File Section 2. Modified REFLECT Scoring Rubric. Supplementary File Section 3. Vignette with Essential Design Elements, Trainee Response, “Rules of Thumb” for Scoring and Qualitative and Qualitative Scoring. Supplementary File Section 4. Steps in Rater Training. [file 12909_2023_4536_MOESM1_ESM.docx]

**Supplementary File Section 1.** Key terms and definitions of reflection*

**REFLECTION**

Reflection is defined as thinking about prior experiences or material already learned that leads to the formulation of additional factual knowledge or conceptual understanding. Reflection can guide practitioners as they encounter the complexity that is inherent to clinical practice, potentially influencing the choice of how to act in “difficult or morally ambiguous circumstances.”

**REFLECTIVE PRACTICE**

Reflective practice is a deliberate way of thinking about prior experiences in order to learn from mistakes. The development of reflective practice has been associated with enhancing an individual's character to identify skills and strengths, and to reflect on options and actions to guide change for future success.

**REFLECTIVE CAPACITY**

Reflective capacity is the ability to critically analyze knowledge and experience in order to achieve deeper meaning and understanding. Fostering reflective capacity within medical education can help develop critical thinking skills, inform clinical reasoning, and enhance professionalism among trainees.

**Essential components to developing reflective capacity**

- noticing the reflective moment
- making sense of the experience (including emotional responses)
- tolerating uncertainty
- using new insights

^*DunnGalvin A, Cooper JB, Shorten G, Blum RH. Applied reflective practice in medicine and anaesthesiology. Br J Anaesth. 2019;122(5):536-541. doi:10.1016/j.bja.2019.02.006^

**Supplementary File Section 2.** Modified REFLECT Scoring Rubric

| **LEVELS of REFLECTION** | Habitual Reflection (non-reflective) | Thoughtful Action or introspection | Reflection | Critical Reflection |  |
| --- | --- | --- | --- | --- | --- |
|  | **1** | **2** | **3** | **4** |  |
| **CRITERION** |  |  |  |  | **Score for each criterion** |
| Presence | No real sense of the writer being present | Sense of the writer being partially present | Sense of the writer being largely present | Sense of the writer being fully present |  |
| Description of conflict/ disorientating dilemma | Little or no description of the conflict or disorienting dilemma (i.e. just describes what happened in basic terms) | Weak description of the conflict or disorienting dilemma with superficial, little or no attention to multiple perspectives, exploring alternative explanations, and challenging assumptions | Good description of the conflict or disorienting dilemma (that can include some attention to others perspective, alternative explanations, or challenging assumptions/status quo) | Very good description of the conflict or disorienting dilemma that includes multiple perspectives, exploring alternative explanations, and challenging assumptions/status quo) |  |
| Attending to emotions | Little or no recognition or attention to emotions | Recognition but little exploration or attention to emotions | Recognition, exploration and attention to emotions AND some emotional insight | Recognition, exploration and attention to emotions AND strong emotional insight |  |
| Analysis and meaning making | Little or no analysis or meaning making | Some analysis or meaning making | Good analysis or meaning making | Very good or comprehensive analysis or meaning making |  |
| Writing spectrum | Superficial descriptive writing approach (fact reporting, vague impressions) without much reflection or introspective) | Elaborated descriptive writing approach and impressions | Reflective writing style that includes movement beyond reporting or descriptive writing to reflecting (i.e. attempting to understand, question or analyse the event) | Critical reflective writing style that includes exploration and critique of assumptions, values, beliefs and/or biases, and the consequences of action (present and future) |  |
|  | | | | | |
| **Total Score LEVEL of REFLECTION** | | | | |  |

**Supplementary File Section 3.** Vignette with Essential Design Elements, Trainee Response, “Rules of Thumb” for Scoring and Qualitative and Qualitative Scoring

REFLECT Vignette - Wet Tap

- **Instructions to Participants**

*Imagine you were the resident in this situation, at any level of training you think it would have been for you. Where we haven’t included details, please feel free to imagine them and incorporate them in your impressions of the clinical situation (e.g., specific conversations or wordings, tone of voice, prior relationships, etc.). The events in this case are based on an actual clinical event. Describe any thoughts and feelings you might have had during and after the event.*

You are doing your first Obstetric Anesthesia rotation and are called to evaluate and place an epidural in a 20-year-old G1P0 parturient female. Review of her medical history is unrevealing, so you obtain consent. Your attending urges you to get started. You have prior experience with epidurals, having placed a few on a General Surgery rotation previously.

You position the patient and set up your tray. After a timeout, you identify your landmarks and then prep and drape in the normal fashion. The patient jumps a bit with the local needle. Your eyes dart toward your attending who is standing on the other side of the patient, closer to the door. He is talking with the nurse but catches your eye and gives you a hand gesture to keep going. You place the Touhy to 2 cm and immediately get some blood return. You mention to your attending, “I got a little heme at 2 cm,” and he says, “No problem just keeping going.”

You engage the syringe with saline and test for loss before advancing. The syringe turns out to not be fully engaged in the Touhy, so you end up spraying saline all over. You refill the syringe and proceed, making sure that the syringe is engaged and also making sure to not press so hard. You check every few millimeters, but there is no loss, so you keep advancing. After a little while, your attending comes to your side of the patient to look. He says, “Wow, I see you are already at 8 cm. Why don’t you clear your Touhy with your stylet?”  As soon as you do this, clear fluid starts dripping back. The attending asks you to withdraw the needle and place it at a different level, which you do under guidance. Loss of resistance is felt at 4 cm and a functional epidural is placed. Your attending discloses the wet tap to the patient and says that there is a fair chance she will have a headache and need a blood patch.

In follow up with the patient, you learn that the patient had a horrible post-dural puncture headache, requiring two blood patches and a week of inpatient stay.

1. What happened?

- The Tuohy needle was blocked, and so I tapped the patient. I then put a working epidural in. The patient got a bad PDPH and needed two blood patches and had to stay in hospital for a week.

1. What are your thoughts and feelings about why this event happened?

- In spite of being very careful, one mistake (not clearing the Tuohy needle of blood) resulted in a PDPH. It was due to lack of experience.
- On the part of the attending, he was supervising me and assumed I would know to clear the Tuohy of blood, but he should have checked.

1. What could you have done differently?

- I could have known to clear the Tuohy needle and I could have insisted that my attending come over and have a look directly at what I was doing.

1. To what extent do you think your written responses about your thoughts and

feelings (not specific clinical actions) are similar to how you’d respond if this were your actual clinical case?

Not at all similar                                    Very similar

1 2 3 4 5 6 7

| **Essential Design Elements** |  |
| --- | --- |
| Background/Setting | A trainee undertaking first Obstetric Anesthesia rotation is asked to evaluate and place an epidural in a 20-year-old G1P0 parturient female. |
| Procedure/Anesthesia Plan | Epidural using a Touhy needle. |
| Main Proponent Actions/Behaviors | The syringe not fully engaged in the Touhy, releasing a considerable amount of saline. |
| Challenge/Conflict/Dilemma/Confrontation | The attending is distracted. |
| Consequences | The patient likely requires a wet tap. |
| Follow up (if relevant) | The patient experiences a very painful post-dural puncture headache, requiring two blood patches and a week of inpatient stay. |

Scoring Guidelines: “Rules of Thumb” for Scoring

***Good reflective writing should have…***

**Presence:** Evidence of presence is prevalent in the text with multiple instances of writing from “I’’ and includes details/inferences/interpretations not necessarily included in the text.

**Description of conflict or disorienting dilemma:** The disorienting dilemma is clearly formulated.

**Attending to emotions:** Emotions are reported, and emotional insight is gained/demonstrated.

**Analysis and meaning making:** The author analyses event through both emotional and cognitive lenses and more than one perspective is included.

**Writing spectrum:** The overall style is reflective or critically reflective, e.g., a detailed and compelling description.

Qualitative and Qualitative Scoring

**Rationale for scoring by the Rater (Qualitative)**

- **Presence (2):** Sense of the writer being partially present. Note the use of “I,” such as*: “I tapped the patient’’* and “*I then put a working epidural in.”* However, there was also a sense of objectivity and distance, e.g., “It was due to lack of experience.” There was also a sense of no personal impacts noted, such as worry, embarrassment, frustration, confusion, pain, with regard to self or patient.
- **Description of conflict or disorienting dilemma (2):** Weak description of the conflict or disorienting dilemma (i.e. The participant just describes what happened in basic terms): “*The Tuohy needle was blocked, and so I tapped the patient. I then put a working epidural in. The patient got a bad PDPH and needed two blood patches and had to stay in hospital for a week.’’*
- **Attending to emotions (1):** Little or no recognition or attention to emotions.
- **Analysis and meaning making (2):** Some analysis and meaning making. The author analyses his suboptimal performance only through a cognitive lens, e.g., *“In spite of being very careful, one mistake (not clearing the Tuohy needle of blood) resulted in a PDPH. It was due to lack of experience’’* and *“I could have insisted that my attending come over and have a look directly at what I was doing.’’*
- **Writing Spectrum (1):** Superficial descriptive writing approach (fact reporting, vague impressions) without much reflection or introspection.

**Rater’s Score (Quantitative)**


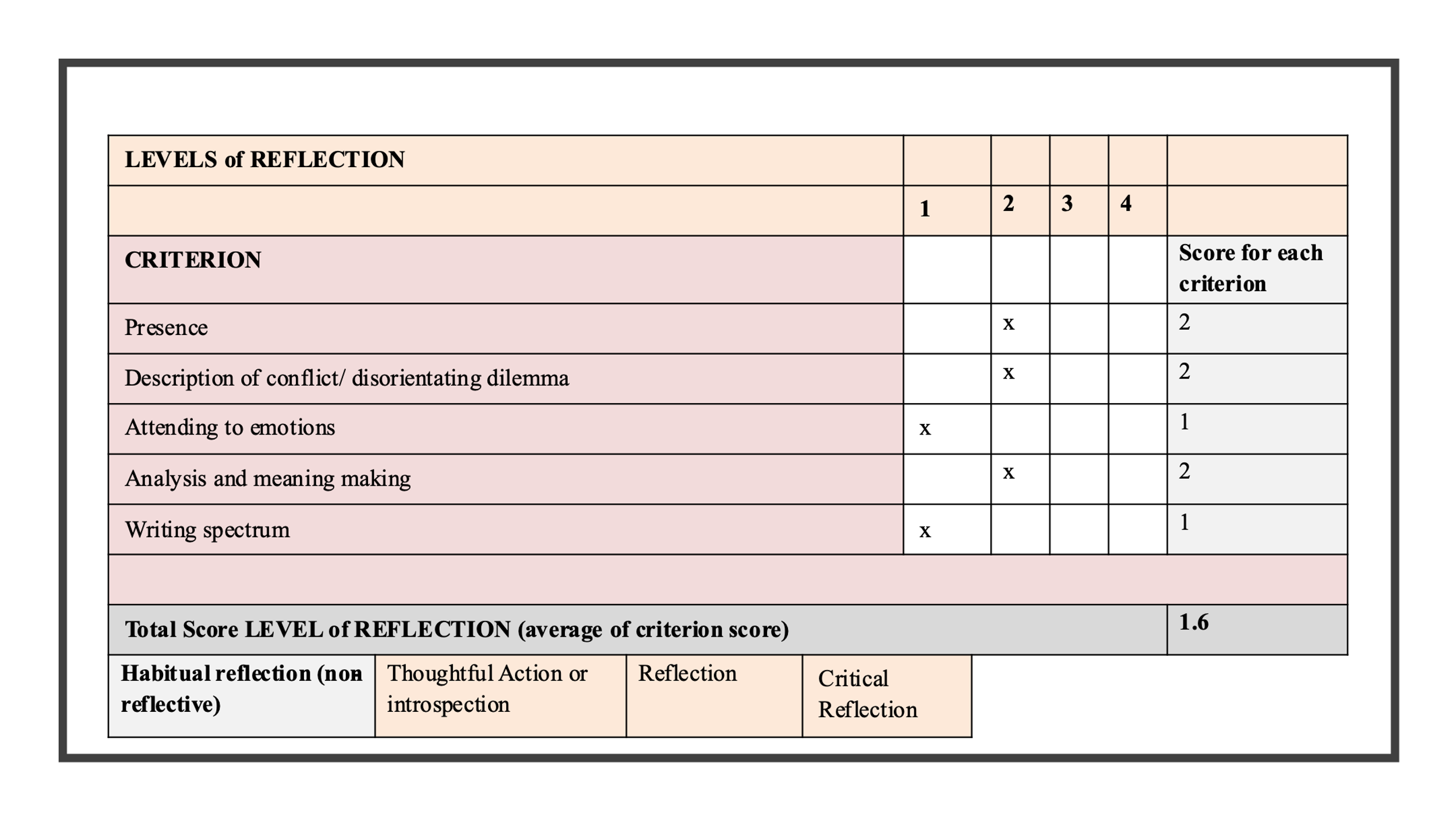


**Supplementary File Section 4:** Steps in Rater Training

Step 1: The trainees were familiarized with the theories underlying the REFLECT Score and scoring.

Step 2: The instructor provided examples of responses of different levels of reflection (and the elements on which each is scored) to illustrate correct scoring.

Step 3: Trainees each scored exemplar vignettes (already rated by instructor) separately and discussed discrepancies in follow up training session. Facilitated discussion continued until agreement across trainees and instructor was <0.5 on a 10-point scale on vignette scoring.

Step 4: Instructor and Trainees each scored 3 novel vignettes drawn from the pool of 9, which were developed based on a set of agreed essential elements (Supplementary File 3).

During training, we used Wald’s four-step process for applying the REFLECT rubric a) Read the response entirely, b) Zoom into details of the response through fragmentation into sentences and phrases to evaluate the presence and quality of each criteria, c) Zoom out to the level of the theme of the whole response taking into consideration the analysis in step 2, d) Be sure to base your score (be able to defend) on examples from the response text.
